# Purification of Chlorophenol Isomers by Stripping Crystallization Combining Melt Crystallization and Vaporization

**DOI:** 10.3390/molecules26216524

**Published:** 2021-10-28

**Authors:** Lie-Ding Shiau

**Affiliations:** 1 Department of Chemical and Materials Engineering, Chang Gung University, Taoyuan 333, Taiwan; shiau@mail.cgu.edu.tw; Tel.: +886-3-2118800 (ext. 5291); Fax: +886-3-2118700; 2 Department of Urology, Linkou Chang Gung Memorial Hospital, Taoyuan 333, Taiwan

**Keywords:** crystallization, vaporization, purification, chlorophenol, thermodynamics process

## Abstract

Stripping crystallization (SC) was introduced in this work to purify *p*-chlorophenol from the *p*-chlorophenol-rich liquid mixture and to purify *m*-chlorophenol from the *m*-chlorophenol-rich liquid mixture, respectively. Essentially, SC combines melt crystallization and vaporization to produce the solid product and the vapor from a liquid mixture via a series of three-phase transformations at reduced pressures during the cooling process. At the end of the SC, only the solid product remained while the liquid mixture was almost eliminated and the produced vapor was removed. A set of differential equations based on the mass and energy balances were proposed to determine the incremental variations of the amounts of remaining liquid, produced solid and produced vapor during the batch SC process. The experimental yield and product purity of the final product obtained from the batch SC experiments were compared with those predicted by the model.

## 1. Introduction

*m*-Chlorophenol and *p*-chlorophenol are important intermediates in the manufacture of solvents, pesticides, textile additives, and specialty chemicals [1]. Due to the close boiling points of *m*-chlorophenol (214 °C) and *p*-chlorophenol (220 °C), it is very difficult to separate them by conventional distillation. A conceptually feasible adductive crystallization using aniline or tert-butanol as an adductive agent has been proposed to separate these two compounds [2,3].

Stripping crystallization (SC) was first introduced by Cheng and Cheng [4] to separate the mixtures of volatile compounds at reduced pressures via a series of three-phase transformations. Based on the concept of SC, Shiau and his coworkers have designed various experimental apparatuses to separate some mixtures with close boiling temperatures, including the mixed xylenes [5,6,7,8,9] and the benzene/cyclohexane mixtures [10] operated within low-temperature ranges, and the ibuprofen enantiomers [11] and 2-amino-1-phenylethanol enantiomers [12] operated within high-temperature ranges. In addition, Shiau and his coworkers proposed various theoretical models to describe the three-phase transformations during the SC process [5,6,7,8,9,10,11,12]. In principle, SC combines melt crystallization and vaporization at reduced pressures. As opposed to the solid-liquid transformations involved during melt crystallization [13,14,15,16,17,18,19,20,21,22,23,24], a series of three-phase transformations occur in a liquid mixture during SC. Consequently, the desired component is crystallized as the solid product while the unwanted components are vaporized and removed. At the end of SC, the liquid mixture disappears and the solid crystalline form of the desired component is obtained.

In the present study, SC was conducted to purify the *m*-Chlorophenol/*p*-chlorophenol liquid mixture. In the first part, SC was applied to purify *p*-chlorophenol from the *p*-chlorophenol-rich liquid mixture. In the second part, SC was applied to purify *m*-chlorophenol from the *m*-chlorophenol-rich liquid mixture. A new model based on the mass and energy balances was proposed to determine the incremental variations of the amounts of remaining liquid, produced solid and produced vapor during the batch SC process.

## 2. SC Models

**Figure 1:**
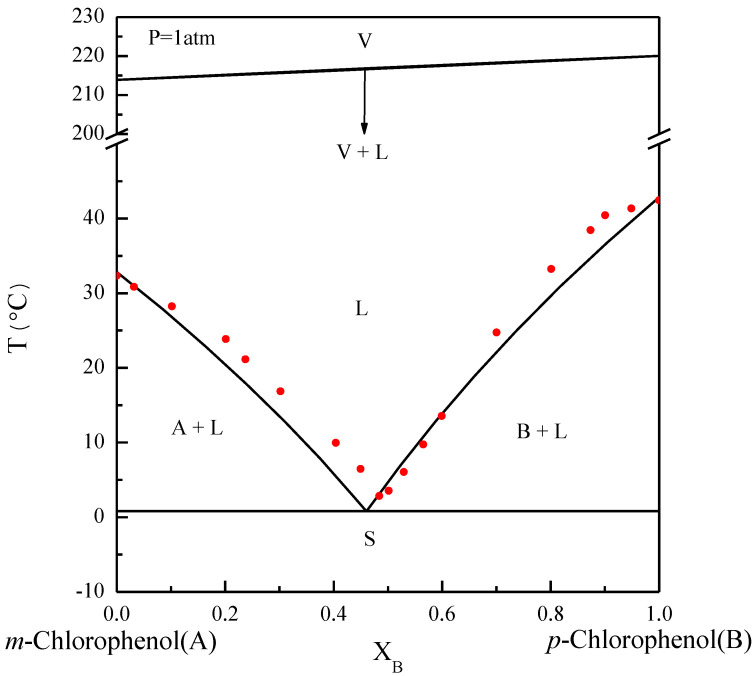
The phase diagram of *m*-chlorophenol (A-component) and *p*-chlorophenol (B-component) at P=1 atm. The solid circle data point represents the experimental SLE reported by Her et al. [2].

The basic principles of the SC process can be explained by referring to phase diagrams. Figure 1 illustrates the phase diagram of *m*-chlorophenol (A-component) and *p*-chlorophenol (B-component) at normal pressure, where the solid–liquid equilibrium (SLE) predicted by the van’t Hoff equation [25,26] is close to the experimental data reported by Her et al. [2]. Note that the eutectic point lies at $X_{B}=0.46$ and T=0.82 °C. Due to the close boiling points of *m*-chlorophenol and *p*-chlorophenol, the bubble points and the dew points for the vapor–liquid equilibrium (VLE) predicted by Raoult’s law [25,26] nearly coincide. Some of the physical properties of *m*-chlorophenol and *p*-chlorophenol are listed in Table 1 [27].

As the pressure is reduced, the SLE boundary typically remains nearly unchanged. However, the bubble temperatures and dew temperatures for the VLE decrease. As the triple-point pressure of the *p*-chlorophenol is 88.7 Pa, the triple-point pressure of the *m*-chlorophenol/*p*-chlorophenol mixture should lie below that and can be determined as follows. 

When SC is applied to produce *p*-chlorophenol solid from the liquid mixture in the range $0.46<X_{B}<1$, a series of three-phase transformations are achieved by lowering the temperature and pressure. Thus, both the SLE and VLE equations need to be satisfied during the SC. The SLE between the *p*-chlorophenol solid and the mixture liquid can be described by the van’t Hoff equation as [25,26]

(1) $$ln\left[X_{B}\left(T\right)\right]=\frac{\Delta H_{m,B}}{R}\left(\frac{1}{T_{m,B}}-\frac{1}{T}\right)\;\;$$

The VLE between the mixture liquid and the mixture vapor can be described by Raoult’s law as [25,26]

(2) $$Y_{A}\left(T\right)P\left(T\right)=X_{A}\left(T\right)P_{A}^{sat}\left(T\right)\;\;\;$$

(3) $$Y_{B}\left(T\right)P\left(T\right)=X_{B}\left(T\right)P_{B}^{sat}\left(T\right)\;\;\;\left(3\right)$$

where $P_{A}^{sat}\left(T\right)$ and $P_{B}^{sat}\left(T\right)$ are the temperature-dependent saturated pressure for *m*-chlorophenol and *p*-chlorophenol, respectively, which are given by the Antoine equation in Table 2. Note that $X_{A}\left(T\right)+X_{B}\left(T\right)=1$ and $Y_{A}\left(T\right)+Y_{B}\left(T\right)=1$. The ideal liquid solution is assumed for simplicity due to the structure similarity between *m*-chlorophenol and *p*-chlorophenol. Due to low pressures, the ideal gas law is assumed for the vapor [25,26].

Combining Equations (2) and (3) yields

(4) $$P\left(T\right)=X_{A}\left(T\right)P_{A}^{sat}\left(T\right)+X_{B}\left(T\right)P_{B}^{sat}\left(T\right)\;$$

**Figure 2:**
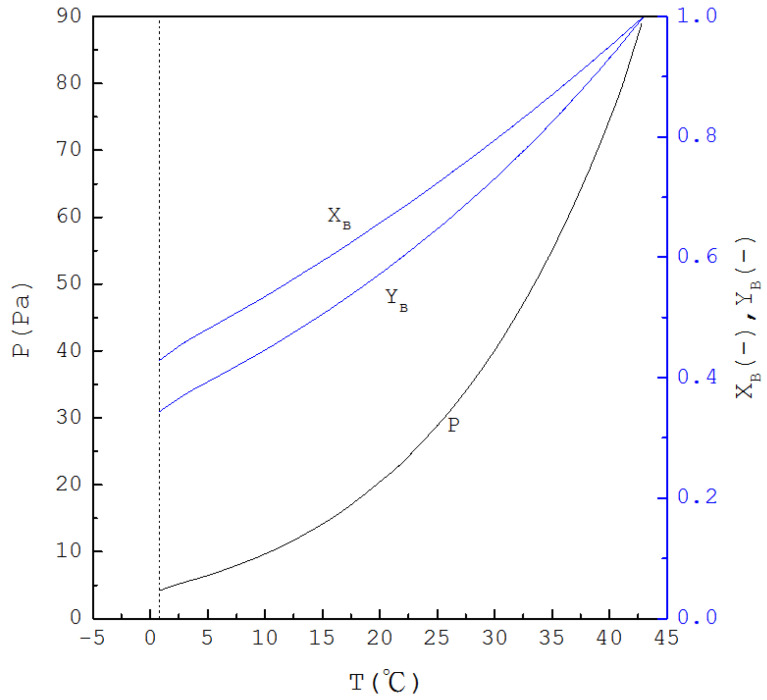
The calculated results of P(T), $X_{B}\left(T\right)$ and $Y_{B}\left(T\right)$ for the formation of *p*-chlorophenol solid product from the liquid mixture via the three-phase transformations.

Thus, if T is specified, $X_{B}\left(T\right)$ is determined from Equation (1) and one has $X_{A}\left(T\right)=1-X_{B}\left(T\right)$; subsequently, as P(T) is determined from Equation (4), $Y_{A}\left(T\right)$ and $Y_{B}\left(T\right)$ are determined from Equations (2) and (3), respectively. Figure 2 displays the calculated three-phase transformation results for the formation of the *p*-chlorophenol solid product, where P(T), $X_{B}\left(T\right)$ and $Y_{B}\left(T\right)$ decrease with decreasing temperature. Thus, as $X_{B}\left(T\right)$ for the mixture liquid decreases due to the formation of the *p*-chlorophenol solid product, the corresponding temperature and pressure for a series of three-phase transformations decrease.

**Figure 3:**
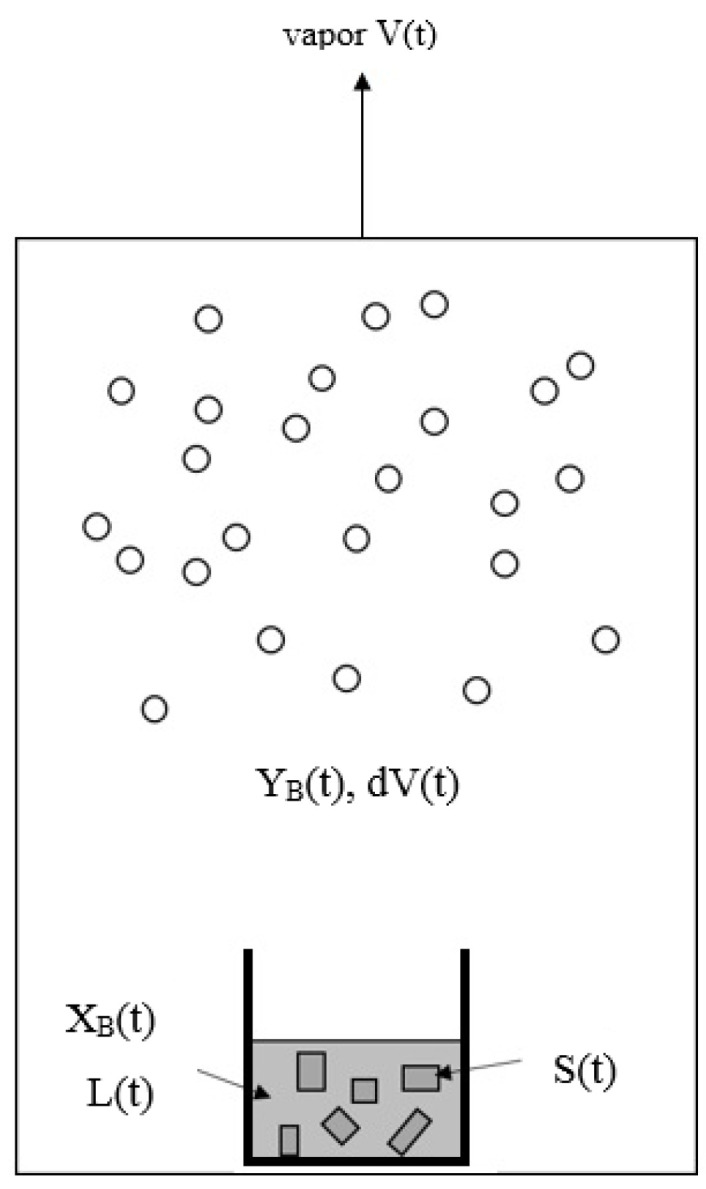
Schematic diagram of SC for a liquid mixture during a series of three-phase transformations, where the vapor formed is removed while the solid product and the remaining liquid is kept in the sample container. The liquid is stirred by a magnetic bar.

Similarly to the decreasing operating temperature for a series of solid–liquid transformations during the batch melt crystallization, the operating temperature is decreased for a series of three-phase transformations during the batch SC process. The batch SC experiment for initially a liquid mixture can be illustrated in Figure 3, where the operating pressure in the chamber needs to be decreased according to P(T) in Figure 2 during the cooling process. As a series of solid–liquid transformations occur during the SC, the liquid mixture disappears gradually, leading to the gradual formation of the *p*-chlorophenol solid product and mixture vapor. The produced *p*-chlorophenol solid product and remaining mixture liquid are kept in the sample container during the SC while the vapor formed is removed from the chamber to keep the operating pressure at P(T).

**Figure 4:**
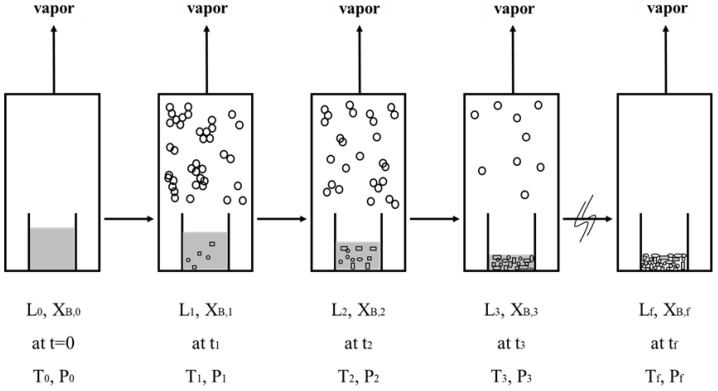
Schematic diagram of a batch SC experiment, where each stage corresponds to a three-phase transformation state at a given time: at t=0, a liquid mixture in the sample container; at $0<t<t_{f}$, formation of the solid product and mixture vapor from a liquid mixture due to the three-phase transformation; at $t_{f}$, only the solid product and the remaining liquid contained in the sample container. Note that the vapor is condensed and collected outside the sample container in the chamber.

For the initial liquid mixture $L_{0}$ with an initial concentration $X_{B,0}$, the initial three-phase transformation temperature and pressure $\left(T_{0,\;\;}\;P_{0}\right)$ can be determined as follows: (a) $T_{0}$ is determined from $X_{B,0}$ based on Equation (1) and (b) $P_{0}$ is determined from $X_{B,0}$ and $T_{0}$ from Equation (4). Figure 4 displays a schematic diagram of the batch SC process for a liquid mixture during a series of three-phase transformations. Each stage corresponds to a three-phase transformation at a given time, $t_{n}$, during the batch experiment. The mixture liquid is fed into the sample container at t=0. As the SC is operated at $0<t<t_{f}$, a series of three-phase transformations occur in the mixture liquid by controlling the temperature and pressure in the chamber. At the conclusion of the batch experiment $\left(t_{f}\right)$, only the *p*-chlorophenol solid product and remaining liquid are contained in the sample container. The mixture vapor formed in each stage is removed.

Both crystallization and vaporization should be kinetic processes. A slower cooling rate during the SC implies a longer operation time at each stage shown in Figure 4, and subsequently a higher likelihood of achieving equilibrium at each stage. On the other hand, a faster cooling rate during the SC implies a shorter operation time at each stage, and subsequently the kinetic processes of crystallization and vaporization need to be considered and equilibrium may not be achieved at each stage. As the SC process in this study was operated at a slow cooling rate, i.e., 0.9 K/min, it was observed during the batch experiments that crystallization and vaporization occurred simultaneously very quickly at each three-phase transformation condition. Thus, it is speculated that equilibrium was achieved at each stage. For simplicity, the SC process for a slow cooling rate can be discussed in terms of a series of three-phase transformations as follows. 

In the previous SC model proposed by Shiau and his coworkers [5,6,7,8,9,10,11], the material and energy balances during the batch SC process were developed based on the stage operation shown in Figure 4. Consequently, the calculated results slightly varied by the stage number selected in the model. However, as temperature and pressure are continuously lowered during the batch SC process shown in Figure 3, a new model is proposed in this work to determine the time variations of the mass of the remaining liquid L(t), the mass of the produced *p*-chlorophenol solid S(t), and the mass of the produced vapor V(t) during the batch SC process. The entire material balance during the batch SC process can be described by

(5) $$-\frac{dL\left(t\right)}{dt}=\frac{dS\left(t\right)}{dt}+\frac{dV\left(t\right)}{dt}\;\;$$

where the left-hand side represents the disappearance rate of liquid while the right-hand side represents the formation rate of solid and vapor.

The material balance of *p*-chlorophenol during the batch SC process can be expressed as

(6) $$-\frac{d\left[L\left(t\right)X_{B}\left(t\right)\right]}{dt}=\frac{dS\left(t\right)}{dt}+Y_{B}\left(t\right)\frac{dV\left(t\right)}{dt}\;$$

where the left-hand side represents the disappearance rate of *p*-chlorophenol in liquid while the right-hand side represents the formation rate of *p*-chlorophenol in solid and vapor. It should be noted that the incremental amount of vapor dV(t) with the concentration $Y_{B}\left(t\right)$ is in VLE with the well-mixed liquid L(t) with the concentration $X_{B}\left(t\right)$. As no impurity trapping is assumed to occur in the formation of the *p*-chlorophenol solid product, S(t) only consists of *p*-chlorophenol.

It was observed during the batch SC experiments that the three-phase transformation occurred very quickly in the mixture liquid, leading to the formation of the *p*-chlorophenol solid product and mixture vapor. Therefore, it is assumed that the heat released in forming the *p*-chlorophenol solid product was quickly removed by vaporizing some portion of the mixture liquid during the SC. As dS(T)/dt represents the formation rate of the solid and dV(T)/dt represents the formation rate of the vapor, the energy balance during the batch SC process can be described as

(7) $$\left[Y_{A}\left(t\right)\Delta H_{V,A}+Y_{B}\left(t\right)\Delta H_{V,B}\right]\frac{dV\left(t\right)}{dt}=\Delta H_{m,B}\;\frac{dS\left(t\right)}{dt}$$

where $Y_{A}\left(t\right)\Delta H_{V,A}+Y_{B}\left(t\right)\Delta H_{V,B}$ corresponds to the heat of vaporization for the produced mixture vapor dV(t) while $\Delta H_{m,B}$ is the heat of crystallization for the produced *p*-chlorophenol solid dS(t).

Depending on the cooling rate adopted during the batch SC process, the operating temperature can be related to the operating time using a cooling function T=f(t) with $T=T_{0}$ at t=0. Thus, one obtains dT=f′(t)dt, where f′(t) is the first derivative of f(t). For example, if the batch SC process is cooled at a constant cooling rate b, one has $T=f\left(t\right)=T_{0}-bt$, leading to f′(t)=−b. By substituting dt with dT/f′(t), combining Equations (5)–(7) regardless of the functional form of f(t) yields

(8) $$\frac{dL\left(T\right)}{dT}=\frac{1}{\frac{\left[Y_{B}\left(T\right)+Y_{A}\left(T\right)f_{A}+Y_{B}\left(T\right)f_{B}\right]}{\left[1+Y_{A}\left(T\right)f_{A}+Y_{B}\left(T\right)f_{B}\right]}-X_{B}\left(T\right)}\frac{X_{B}\left(T\right)\Delta H_{m,B}L\left(T\right)}{RT^{2}}$$

(9) $$\frac{dV\left(T\right)}{dT}=-\frac{1}{1+Y_{A}\left(T\right)f_{A}+Y_{B}\left(T\right)f_{B}}\frac{dL\left(T\right)}{dT}\;\;$$

(10) $$\frac{dS\left(T\right)}{dT}=-\frac{dL\left(T\right)}{dT}-\frac{dV\left(T\right)}{dT}$$

where $f_{A}=\Delta H_{V,A}/\Delta H_{m,B}$ and $f_{B}=\Delta H_{V,B}/\Delta H_{m,B}$. Note that Equation (1) leads to $dX_{B}\left(T\right)/dT=X_{B}\left(T\right)\Delta H_{m,B}/RT^{2}$. Thus, if T is specified, $X_{B}\left(T\right)$, $Y_{A}\left(T\right)$ and $Y_{B}\left(T\right)$ can be determined previously from Equations (1)–(4). Consequently, Equations (8)–(10) constitute a set of differential equations that can be numerically solved for S(T), L(T), and V(T) during the batch SC process. Initially, one has $L\left(T_{0}\right)=L_{0}$, $X_{B}\left(T_{0}\right)=X_{B,0}$ and $S\left(T_{0}\right)=V\left(T_{0}\right)=0$ at the beginning of the SC. It should be noted that the above model is not applicable if the SC process is operated at a fast cooling rate, where the kinetic processes of crystallization and vaporization need to be considered and equilibrium may not be achieved at each stage.

**Figure 5:**
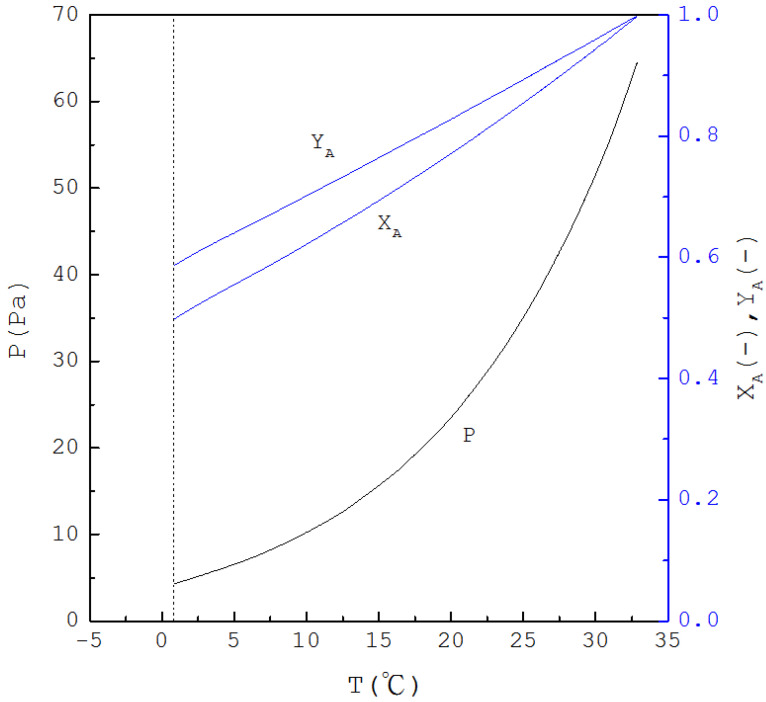
The calculated results of P(T), $X_{A}\left(T\right)$ and $Y_{A}\left(T\right)$ for the formation of *m*-chlorophenol solid product from the liquid mixture via the three-phase transformations.

Similar equations can be derived when SC is applied to purify *m*-chlorophenol (A-component) from the liquid mixture in the range $0<X_{B}<0.46$ (see Appendix A). Figure 5 displays the calculated three-phase transformation results for the formation of *m*-chlorophenol solid product, where P(T), $X_{A}\left(T\right)$ and $Y_{A}\left(T\right)$ decrease with decreasing temperature. Thus, as $X_{A}\left(T\right)$ for the mixture liquid decreases due to the formation of the *m*-chlorophenol solid product, the corresponding temperature and pressure for a series of three-phase transformations decrease.

## 3. Experimental Section

**Figure 6:**
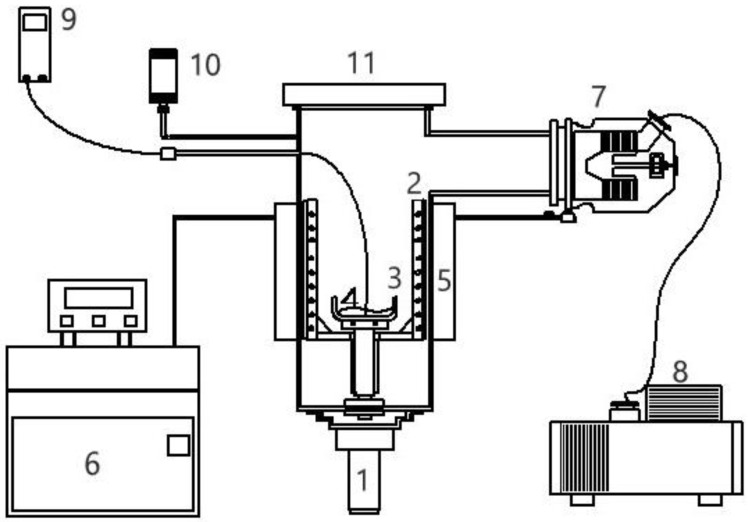
Schematic diagram of the experimental apparatus for SC with the features: (1) magnetic-driven motor, (2) rotating scraper, (3) sample container, (4) sample, (5) coolant jacket, (6) cooling system, (7) turbomolecular pump, (8) mechanical pump, (9) thermocouple, (10) pressure gauge, (11) transparent cover.

The SC experiments were performed using the experimental assembly in Figure 6, which consists of 0.5-L sample vessel in a 10-L chamber. Crystallization and vaporization of the mixture liquid during the three-phase transformation was observed in the chamber via transparent cover. *m*-Chlorophenol (purity >99%) and *p*-chlorophenol (purity >99%) were purchased from ACROS. The whole chamber was fitted with a cooling jacket to lower the temperature in the chamber. A mechanical vacuum pump and turbomolecular pump were used in series to lower the pressure in the chamber. A pressure gauge was connected to the large chamber and a temperature probe was positioned in the mixture liquid.

To perform SC for 20 g liquid feed with an initial concentration, the initial corresponding three-phase transformation temperature and pressure $\left(T_{0},P_{0}\right)$ were determined first. Depending on the initial concentration, the initial three-phase transformation generally occurred slightly below the triple-point of *p*-chlorophenol $\left(T_{tri}=43\;{}^{\circ}C,P_{tri}=88.7\;\mathrm{Pa}\right)$ for the purification of *p*-chlorophenol or below the triple-point of *m*-chlorophenol $\left(T_{tri}=33\;{}^{\circ}C,P_{tri}=63.1\;\mathrm{Pa}\right)$ for the purification of *m*-chlorophenol. Then, P(T) in Figure 2 for the purification of *p*-chlorophenol or P(T) in Figure 5 for the purification of *m*-chlorophenol was adopted to direct the experiments for a series of three-phase transformation conditions during the cooling process.

At the beginning of the experiment, 20 g mixture liquid was injected into the sample container stirred by the magnetic-driven motor at 120 rpm. The chamber was cooled at a constant cooling rate of 0.9 K/min. Thus, the cooling function corresponds to $T=f\left(t\right)=T_{0}-0.9t$. As the temperature decreased in the chamber, the corresponding operating pressure was reduced by controlling the vacuum pump in keeping with the three-phase transformation conditions. Consequently, a series of three-phase transformations occurred in the liquid mixture, resulting in the formation of *p*-chlorophenol (or *m*-chlorophenol) crystalline product and mixture vapor. When the vapor was brought into contact with the cold inner wall of the chamber, the formed solid adhered to the cold surface and interfered with heat transfer. A rotating scraper was equipped to remove the desublimate from the cold inner wall of the chamber.

For each feed, the experiment was started from the corresponding initial three-phase transformation condition $\left(T_{0},P_{0}\right)$ and was ended at the final three-phase transformation condition $\left(T_{f},P_{f}\right)$ when vaporization was no longer observed in the chamber. At the end of the experiment, the sample remained in the vessel, was weighed, and the product purity was determined by GC using a Perkin–Elmer Clarus 500 series gas chromatograph with a stainless-steel capillary column [Bentone 34/DNDP SCOT, 50 ft × 0.02 in. (i.d.), Supelco, USA].

## 4. Results and Discussion

**Figure 7:**
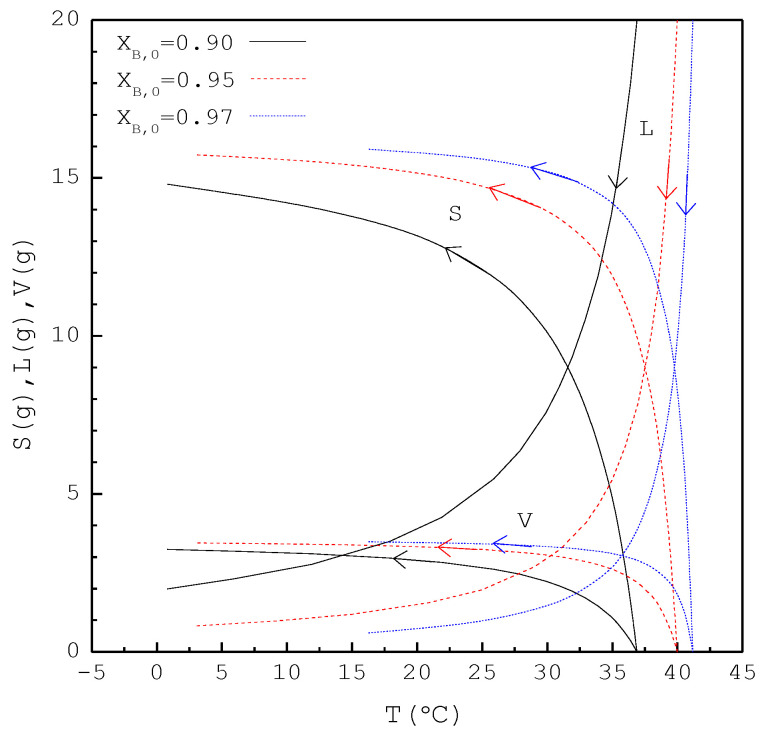
The calculated results of S(T), L(T), and V(T) during the SC cooling process for the purification of *p*-chlorophenol.

In the first part, the SC was applied to purify *p*-chlorophenol from 20 g liquid feed $\left(L_{0}=20\;g,S_{0}=V_{0}=0\right)$ with $X_{B,0}=0.90$, 0.95, and 0.97, respectively. The initial and final three-phase transformation conditions for each $X_{B,0}$ are listed in Table 3. Figure 7 shows the calculated results of S(T), L(T), and V(T) during the cooling process. Note that S increases rapidly during the early cooling process and then increases slowly during the later cooling process; however, L decreases rapidly during the early cooling process and then decreases slowly during the later cooling process. Based on the total material balance during the SC, $L_{0}=L\left(T\right)+S\left(T\right)+V\left(T\right)$ is obtained. It should be noted that L for $X_{B,0}=0.97$ decreases more rapidly during the early cooling process than that for $X_{B,0}=0.90$, and subsequently, S for $X_{B,0}=0.97$ increases more rapidly during the early cooling process than that for $X_{B,0}=0.90$.

As the model predicts, there is some remaining liquid along with the solid at the end of the SC; the final product consists of the final *p*-chlorophenol crystalline product and remaining liquid. The calculated yield for the final product is defined as

(11) $$W_{f,the}=S_{f}+L_{f}\;$$

where $S_{f}$ denotes the calculated mass of the *p*-chlorophenol solid at the end of the SC and $L_{f}$ denotes the calculated mass of liquid at the end of the SC. The calculated purity of the *p*-chlorophenol in the final product is given by

(12) $$Z_{B,the}=\frac{S_{f}+L_{f}X_{B,f}}{S_{f}+L_{f}}\;$$

where $X_{B,f}$ denotes the calculated concentration of the *p*-chlorophenol in liquid at the end of the SC. For comparison, $W_{f,exp}$ refers to the total weight of the final product measured at the end of the experiments and $Z_{B,exp}$ refers to the experimental purity of the *p*-chlorophenol in the final product measured at the end of the experiments. It should be noted that, although $W_{f,exp}$ can be measured at the end of experiments, the experimental final *p*-chlorophenol crystalline product and remaining liquid cannot be respectively measured.

**Figure 8:**
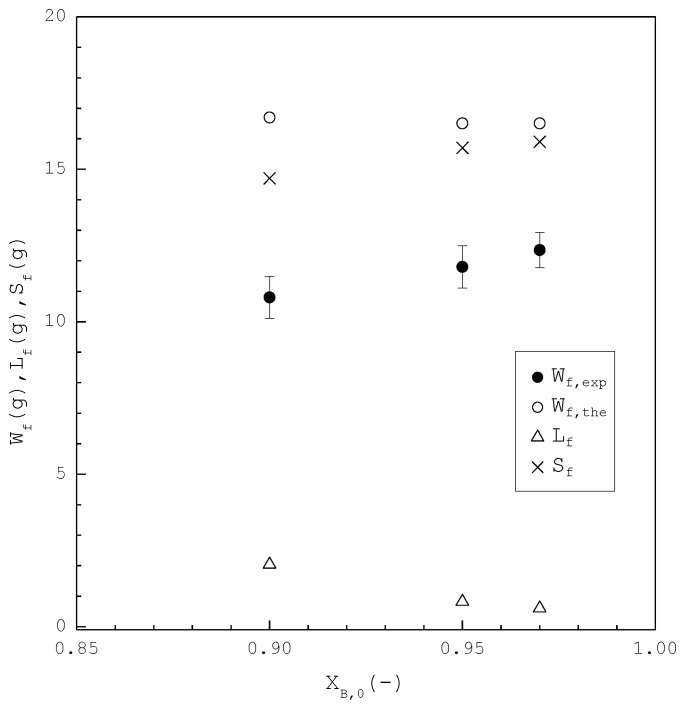
Comparison of $L_{f}$, $S_{f}$, $W_{f,the}$ and $W_{f,exp}$ of the final product plotted against $X_{B,0}$ for the purification of *p*-chlorophenol from 20 g liquid feed. Each solid circle data point represents the average $W_{f,exp}$ for four repetitive experiments and error bar represents the 95% confidence interval for the experimental $W_{f,exp}$.

**Figure 9:**
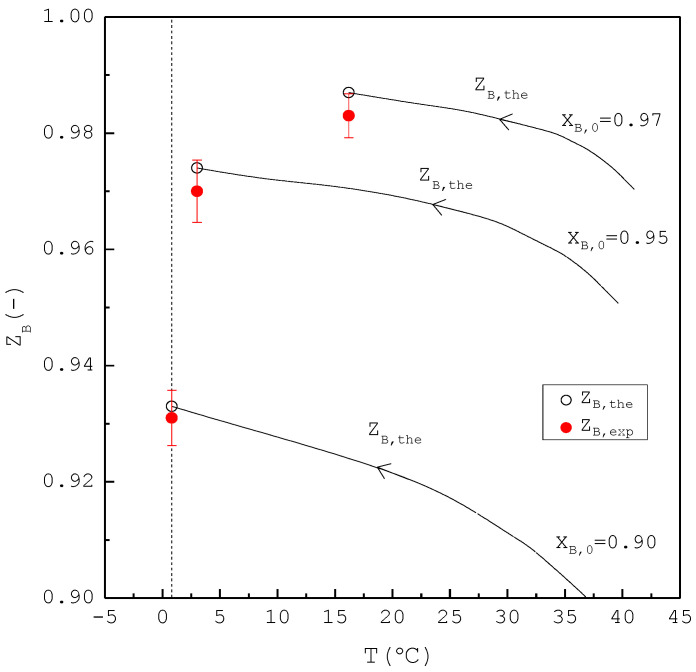
Comparison between $Z_{B,exp}$ and $Z_{B,the}$ of the final product for the purification of *p*-chlorophenol from 20 g liquid feed for each $X_{B,0}$, where $Z_{B,the}\left(T\right)$ is plotted against the operating temperature for each $X_{B,0}$. Each solid circle data point represents the average $Z_{B,exp}$ for four repetitive experiments and error bar represents the 95% confidence interval for the experimental $Z_{B,exp}$.

Figure 8 shows a comparison of $L_{f}$, $S_{f}$, $W_{f,the}$ and $W_{f,exp}$ of the final product against $X_{B,0}$ in the purification of p-chlorophenol. As $X_{B,0}$ increases, $L_{f}$ decreases while $S_{f}$ increases. Consequently, $W_{f,the}$ remains nearly the same regardless of $X_{B,0}$. However, $W_{f,exp}$ is generally smaller than $W_{f,the}$ for each $X_{B,0}$. This is attributed to the fact that the three-phase transformation conditions may not always be attained during the batch experiments. If the operating pressure was lower than the three-phase transformation pressure during the cooling process, the operating condition might favor the vapor formation during the experiments, leading to a smaller $W_{f,exp}$ compared to $W_{f,the}$. Figure 9 compares the $Z_{B,exp}$ and $Z_{B,the}$ in the purification of p-chlorophenol for each $X_{B,0}$, where $Z_{B,the}\left(T\right)$ is plotted against the operating temperature for each $X_{B,0}$. The starting point of $Z_{B,the}\left(T\right)$ represents the feed purity and initial operating temperature; the ending point refers to the calculated product purity and final operating temperature. The results indicate that $Z_{B,exp}$ is close to $Z_{B,the}$ for each $X_{B,0}$.

**Figure 10:**
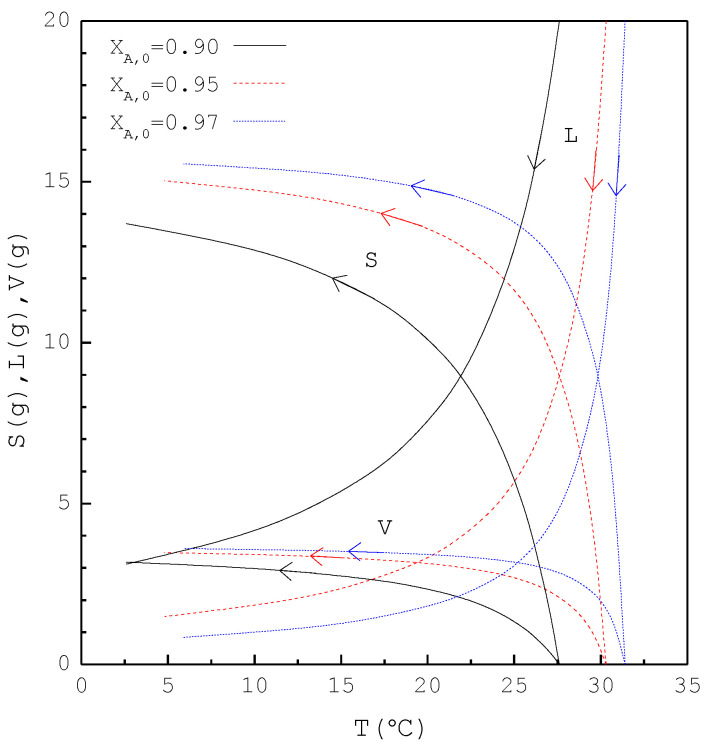
The calculated results of S(T), L(T), and V(T) during the SC cooling process for the purification of m-chlorophenol.

**Figure 11:**
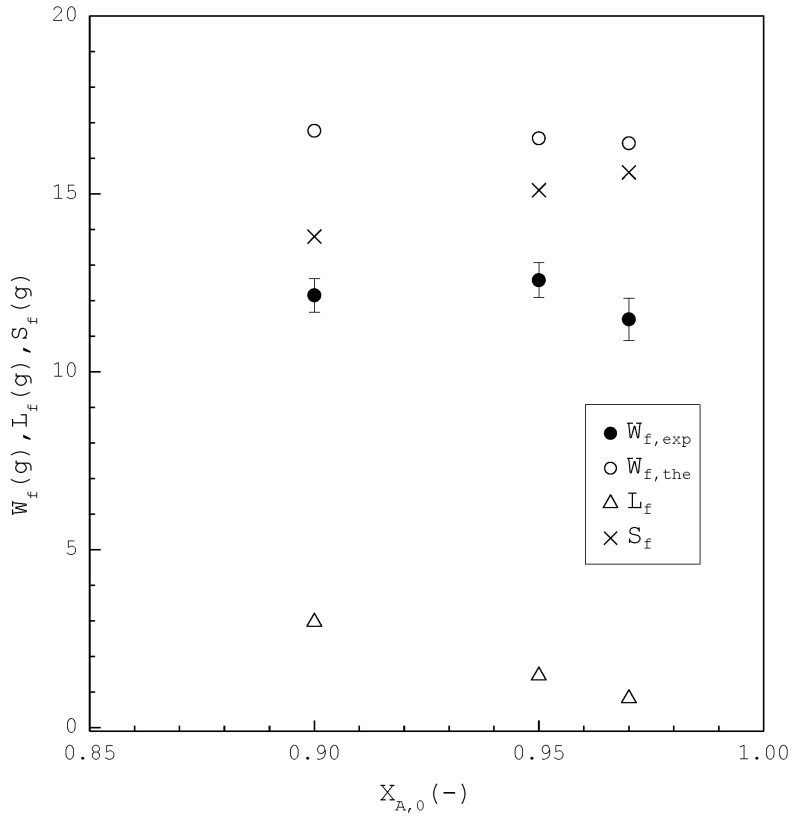
Comparison of $L_{f}$, $S_{f}$, $W_{f,the}$ and $W_{f,exp}$ of the final product plotted against $X_{A,0}$ for the purification of *m*-chlorophenol. Each solid circle data point represents the average $W_{f,exp}$ for four repetitive experiments and error bar represents the 95% confidence interval for the experimental $W_{f,exp}$.

**Figure 12:**
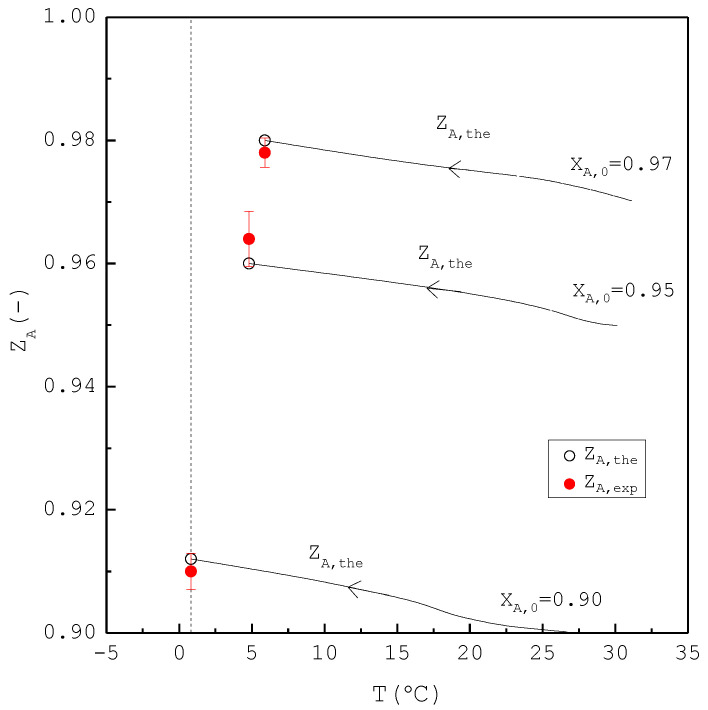
Comparison between $Z_{A,exp}$ and $Z_{A,the}$ of the final product for the purification of *m*-chlorophenol for each $X_{A,0}$, where $Z_{A,the}\left(T\right)$ is plotted against the operating temperature for each $X_{A,0}$. Each solid circle data point represents the average $Z_{A,exp}$ for four repetitive experiments and error bar represents the 95% confidence interval for the experimental $Z_{A,exp}$.

In the second part, the SC was applied to purify *m*-chlorophenol from 20 g liquid feed $\left(L_{0}=20\;g,S_{0}=V_{0}=0\right)$ with $X_{A,0}=0.90$, 0.95, and 0.97, respectively. The initial and final three-phase transformation conditions for each $X_{A,0}$ are listed in Table 4. Figure 10 shows the calculated results of S(T), L(T), and V(T) during the cooling process. Figure 11 compares the $L_{f}$, $S_{f}$, $W_{f,the}$ and $W_{f,exp}$ of the final product against $X_{A,0}$ in the purification of the *m*-chlorophenol. Figure 12 shows a comparison between $Z_{A,exp}$ and $Z_{A,the}$ in the purification of *m*-chlorophenol for each $X_{A,0}$. The results in Figure 10, Figure 11 and Figure 12 for the purification of *m*-chlorophenol are similar to those in Figure 7, Figure 8 and Figure 9 for the purification of *p*-chlorophenol.

As compared in Table 3 and Table 4, the operating temperature range $\left(T_{0}-T_{f}\right)$ in the purification of *p*-chlorophenol is greater than that in the purification of *m*-chlorophenol for $X_{A,0}=X_{B,0}$. Consequently, the model predicts that SC leads to a smaller $L_{f}$ due to a greater operating temperature range in the purification of *p*-chlorophenol than in the purification of *m*-chlorophenol for $X_{A,0}=X_{B,0}$, as shown in Figure 8 and Figure 11. As a smaller $L_{f}$ results in higher purity of the final product, the model predicts that the final *p*-chlorophenol product has a higher purity than the final *m*-chlorophenol, which is consistent with the experimental results. For example, as the SC was operated from $T_{0}=36.9\;{}^{\circ}C$ to $T_{f}=0.82\;{}^{\circ}C$, $X_{B,0}=0.90$ was experimentally purified to $Z_{B,exp}=0.931$ as opposed to $Z_{B,the}=0.932$ with $L_{f}=2.0\;g$ in the purification of *p*-chlorophenol. On the other hand, as the SC was operated from $T_{0}=27.6\;{}^{\circ}C$ to $T_{f}=0.82\;{}^{\circ}C$ in the purification of *m*-chlorophenol, $X_{A,0}=0.90$ was experimentally purified to $Z_{A,exp}=0.910$ as opposed to $Z_{A,the}=0.912$ with $L_{f}=3.0\;g$ in the purification of *m*-chlorophenol. Thus, it was easier to purify *p*-chlorophenol from the *p*-chlorophenol-rich liquid mixture than to purify *m*-chlorophenol from the *m*-chlorophenol-rich liquid mixture.

Discrepancies between the calculated and experimental results of yield and product purity are attributed to: (a) the actual three-phase transformation conditions during the experiments may have deviated from the three-phase transformation conditions determined from Equations (1)–(4); (b) the calculated results from Equations (8)–(10) were obtained based on the assumption that SC was always maintained in a series of three-phase transformation conditions, which may not always have been achieved during the experiments; (c) the calculated results were based on the assumption of no liquid inclusion, which may have occurred during the experiments and (d) the calculated results were based on the assumption of no impurity incorporated into the crystal lattice, which may have occurred during the experiments due to the similar molecular structures of *p*-chlorophenol and *m*-chlorophenol. In this work, the weight and purity were measured at the end of the batch experiments, but in situ process analysis tools could be a good option for future studies to implement with the experimental setup and by which the experimental data can be closely compared with the predicted results from the model. Furthermore, a feedback control system could be applied in future studies to make the states in the crystallizer more closed to the three-phase transformation conditions.

## 5. Conclusions

The SC was successfully applied to purify the *m*-chlorophenol/*p*-chlorophenol liquid mixture. A new model based on the mass and energy balances was proposed to determine the differential variations of the amounts of remaining liquid, produced solid and produced vapor via a series of three-phase transformations during the batch SC process. For both the purification of *p*-chlorophenol and the purification of *m*-chlorophenol, the experimental purity of the final product was close to the calculated purity of the final product, while the experimental yield was lower than the calculated yield. As consistent with the model’s predictions, the experiments indicated for the same concentration of impurity in the liquid feed that a higher purity of the final product was obtained at the end of SC in the purification of *p*-chlorophenol than in the purification of *m*-chlorophenol.

## Figures and Tables

**Table 1:** Some physical properties for *m*-chlorophenol and *p*-chlorophenol [27].

Property	*m*-Chlorophenol	*p*-Chlorophenol
Molecular weight	128.6	128.6
$T_{b}\left({}^{\circ}C\right)$	214	220
$T_{m}\left({}^{\circ}C\right)$	33	43
$P_{tri}\left(\mathrm{Pa}\right)$	63.1	88.7
$\Delta H_{m}\left(J/\mathrm{mol}\right)$	$1.49\times 10^{4}$	$1.41\times 10^{4}$
$\Delta H_{V}\left(J/\mathrm{mol}\right)$	$6.35\times 10^{4}$	$6.44\times 10^{4}$

**Table 2:** The constants of the Antoine equation, $\log_{10}\;\left(\frac{P^{sat}}{1.01\times 10^{5}}\right)=a\;-\frac{b}{c+T}$ ($P^{sat}$ in Pa and T in K), for *m*-chlorophenol and *p*-chlorophenol [27].

Constants	*m*-Chlorophenol	*p*-Chlorophenol
a	4.67081	4.92975
b	2074.632	2278.849
c	−42.359	−30.80

**Table 3:** The initial and final three-phase transformation conditions for the formation of *p*-chlorophenol solid product during the batch SC experiments.

$X_{B,0}$	$T_{0}\;\left({}^{\circ}C\right)$	$P_{0}\;(\mathrm{Pa})$	$T_{f}\;\left({}^{\circ}C\right)$	$P_{f}\;(\mathrm{Pa})$
0.90	36.9	61.8	0.82	4.5
0.95	40.0	74.5	3.0	5.5
0.97	41.2	79.9	16.2	15.4

**Table 4:** The initial and final three-phase transformation conditions for the formation of *m*-chlorophenol solid product during the batch SC experiments.

$X_{A,0}$	$T_{0}\;\left({}^{\circ}C\right)$	$P_{0}\;(\mathrm{Pa})$	$T_{f}\;\left({}^{\circ}C\right)$	$P_{f}\;(\mathrm{Pa})$
0.90	27.6	43.0	0.82	4.4
0.95	30.3	52.8	4.8	6.4
0.97	31.4	57.4	5.9	7.1

## Data Availability

Data is contained within the article.

## Notation

$f_{A}$ = defined as $\Delta H_{V,A}/\Delta H_{m,B}$, dimensionless
$f_{B}$ = defined as $\Delta H_{V,B}/\Delta H_{m,B}$, dimensionless
L = mass of the liquid mixture, g
$L_{0\;}$ = mass of the initial liquid mixture, g
$L_{f\;}$ = mass of the final liquid mixture, g
P = pressure, Pa
$P_{j}^{sat}$ = saturated vapor pressure of component-j, Pa
$P_{tri,j}$ = triple-point pressure of component-j, K
R = ideal gas constant, 8.314 J/mol−K
S = mass of the solid phase, g
$S_{0\;}$ = mass of the initial solid phase, g
$S_{f\;}$ = mass of the final solid phase, g
T = temperature, K
$T_{b,j}$ = boiling temperature of component-j, K
$T_{m,j}$ = melting temperature of component-j, K
$T_{tri,j}$ = triple-point temperature of component-j, K
t = time, min
V = mass of the vapor phase, g
$V_{0\;}$ = mass of the initial vapor phase, g
$W_{f,exp}$ = experimental weight of the final product, g
$W_{f,the}$ = calculated weight of the final product, g
$X_{j}$ = mole fraction of component-j in the liquid mixture, dimensionless
$X_{j,0}$ = initial mole fraction of component-j in the liquid mixture, dimensionless
$X_{j,f}$ = final mole fraction of component-j in the liquid mixture, dimensionless
$Y_{j}$ = mole fraction of component-j in the vapor phase, dimensionless
$Z_{j,exp}$ = experimental purity of component-j in the final product, dimensionless
$Z_{j,the}$ = calculated purity of component-j in the final product, dimensionless
$\Delta H_{m,j\;}$ = heat of melting for component-j (>0), J/mol
$\Delta H_{V,j\;}$ = heat of vaporization for component-j (>0), J/mol

## Subscript

0 = in the initial feed
f = at the end of SC

## Appendix A

When SC is applied to produce the *m*-chlorophenol (A-component) solid product from the liquid mixture in the range $0<X_{B}<0.46$, the SLE between the *m*-chlorophenol solid and the mixture liquid can be described by van’t Hoff equation as [25,26]

(A1) $$ln\left[X_{A}\left(T\right)\right]=\frac{\Delta H_{m,A}}{R}\left(\frac{1}{T_{m,A}}-\frac{1}{T}\right)$$

The VLE between the mixture liquid and the mixture vapor can be described by Raoult’s law as [25,26]

(A2) $$Y_{A}\left(T\right)P\left(T\right)=X_{A}\left(T\right)P_{A}^{sat}\left(T\right)$$

(A3) $$Y_{B}\left(T\right)P\left(T\right)=X_{B}\left(T\right)P_{B}^{sat}\left(T\right))$$

Note that $X_{A}\left(T\right)+X_{B}\left(T\right)=1$ and $Y_{A}\left(T\right)+Y_{B}\left(T\right)=1$. Combining Equations (A2) and (A3) yields

(A4) $$P\left(T\right)=X_{A}\left(T\right)P_{A}^{sat}\left(T\right)+X_{B}\left(T\right)P_{B}^{sat}\left(T\right)$$

Thus, if T is specified, $X_{A}\left(T\right)$ is determined based on Equation (A1) and one has $X_{B}\left(T\right)=1-X_{A}\left(T\right)$; subsequently, as P(T) is determined from Equation (A4), $Y_{A}\left(T\right)$ and $Y_{B}\left(T\right)$ are determined from Equations (A2) and (A3), respectively.

The entire material balance during the batch SC process can be described by

(A5) $$-\frac{dL\left(t\right)}{dt}=\frac{dS\left(t\right)}{dt}+\frac{dV\left(t\right)}{dt}\;$$

The material balance of *m*-chlorophenol during the batch SC process can be expressed as

(A6) $$-\frac{d\left[L\left(t\right)X_{A}\left(t\right)\right]}{dt}=\frac{dS\left(t\right)}{dt}+Y_{A}\left(t\right)\frac{dV\left(t\right)}{dt}\;$$

The energy balance during the batch SC process can be described as

(A7) $$\;\left[Y_{A}\left(t\right)f_{A}+Y_{B}\left(t\right)f_{B}\right]\frac{dV\left(t\right)}{dt}=\frac{dS\left(t\right)}{dt}\;\;\;$$

where $f_{A}=\Delta H_{V,A}/\Delta H_{m,A}$ and $f_{B}=\Delta H_{V,B}/\Delta H_{m,A}$.

By substituting dt with dT/f′(t), combining Equations (A5)–(A7) yields

(A8) $$\frac{dL\left(T\right)}{dT}=\frac{1}{\frac{\left[Y_{A}\left(T\right)+Y_{A}\left(T\right)f_{A}+Y_{B}\left(T\right)f_{B}\right]}{\left[1+Y_{A}\left(T\right)f_{A}+Y_{B}\left(T\right)f_{B}\right]}-X_{A}\left(T\right)}\frac{X_{A}\left(T\right)\Delta H_{m,A}L\left(T\right)}{RT^{2}}$$

(A9) $$\frac{dV\left(T\right)}{dT}=-\frac{1}{1+Y_{A}\left(T\right)f_{A}+Y_{B}\left(T\right)f_{B}}\frac{dL\left(T\right)}{dT}$$

(A10) $$\frac{dS\left(T\right)}{dT}=-\frac{dL\left(T\right)}{dT}-\frac{dV\left(T\right)}{dT}\;$$

Note that Equation (A1) leads to $dX_{A}\left(T\right)/dT=X_{A}\left(T\right)\Delta H_{m,A}/RT^{2}$. Thus, if T is specified, $X_{A}\left(T\right)$, $Y_{A}\left(T\right)$ and $Y_{B}\left(T\right)$ can be determined previously from Equations (A1)–(A4). Consequently, Equations (A8)–(A10) constitute a set of differential equations that can be numerically solved for S(T), L(T), and V(T) during the batch SC process. Initially, one has $L\left(T_{0}\right)=L_{0}$, $X_{A}\left(T_{0}\right)=X_{A,0}$ and $S\left(T_{0}\right)=V\left(T_{0}\right)=0$ at the beginning of SC.
